# Chemotaxis without Conventional Two-Component System, Based on Cell Polarity and Aerobic Conditions in Helicity-Switching Swimming of *Spiroplasma eriocheiris*

**DOI:** 10.3389/fmicb.2017.00058

**Published:** 2017-02-03

**Authors:** Peng Liu, Huajun Zheng, Qingguo Meng, Natsuho Terahara, Wei Gu, Shengyue Wang, Guoping Zhao, Daisuke Nakane, Wen Wang, Makoto Miyata

**Affiliations:** ^1^Jiangsu Key Laboratory for Biodiversity and Biotechnology and Jiangsu Key Laboratory for Aquatic Crustacean Diseases, College of Life Sciences, Nanjing Normal UniversityJiangsu, China; ^2^Department of Biology, Graduate School of Science, Osaka City UniversityOsaka, Japan; ^3^Shanghai-MOST Key Laboratory of Health and Disease Genomics, Chinese National Human Genome Center at ShanghaiShanghai, China; ^4^Department of Physics, Gakushuin UniversityTokyo, Japan; ^5^The OCU Advanced Research Institute for Natural Science and Technology, Osaka City UniversityOsaka, Japan

**Keywords:** MreB, Fibril, reversal behavior, anaerobic, cytoskeleton, genome, mass spectrometry, crab pathongen

## Abstract

*Spiroplasma eriocheiris* is a pathogen that causes mass mortality in Chinese mitten crab, *Eriocheir sinensis*. *S. eriocheiris* causes tremor disease and infects almost all of the artificial breeding crustaceans, resulting in disastrous effects on the aquaculture economy in China. *S. eriocheiris* is a wall-less helical bacterium, measuring 2.0 to 10.0 μm long, and can swim up to 5 μm per second in a viscous medium without flagella by switching the cell helicity at a kink traveling from the front to the tail. In this study, we showed that *S. eriocheiris* performs chemotaxis without the conventional two-component system, a system commonly found in bacterial chemotaxis. The chemotaxis of *S. eriocheiris* was observed more clearly when the cells were cultivated under anaerobic conditions. The cells were polarized as evidenced by a tip structure, swimming in the direction of the tip, and were shown to reverse their swimming direction in response to attractants. Triton X-100 treatment revealed the internal structure, a dumbbell-shaped core in the tip that is connected by a flat ribbon, which traces the shortest line in the helical cell shape from the tip to the other pole. Sixteen proteins were identified as the components of the internal structure by mass spectrometry, including Fibril protein and four types of MreB proteins.

## Introduction

*Spiroplasma* is a genus of bacteria that belongs to the class *Mollicutes*, which includes groups of economically important pathogens, such as the *Mycoplasma* and *Phytoplasma* species. These bacteria are characterized by small genomes and the lack of a peptidoglycan layer (Davis et al., 1972; Daniels et al., 1980; Daniels and Longland, 1984; Trachtenberg, 1998). Spiroplasmas form a helical cell shape and swim without flagella in viscous media (Daniels et al., 1980; Daniels and Longland, 1984; Gilad et al., 2003) by propagation of kink pairs along their cell body from front to back (Gilad et al., 2003; Shaevitz et al., 2005; Wada and Netz, 2007, 2009). The 13 *Spiroplasma* genomes reported so far do not have orthologs of other bacterial motility systems but have five to seven homologs of the protein MreB (Table S1; Ku et al., 2013, 2014; Lo et al., 2015; Davis et al., 2015a,b). MreB is related to actin, which is responsible for many eukaryotic motility systems. The distinct morphology of spiroplasmas is defined by an internal structure, a flat ribbon assembled by antiparallel seven fibrils (Townsend et al., 1980; Williamson et al., 1991; Kürner et al., 2005; Cohen-Krausz et al., 2011). Although two filamentous proteins have been identified including Fibril and MreB (Williamson et al., 1991; Trachtenberg et al., 2008), the whole image of the internal structure is still unclear.

During chemotaxis, most motile bacteria change the reversal frequency of movements through a two-component system (TCS) (Adler, 1974; Porter et al., 2011; Typas and Sourjik, 2015). *Spiroplasma melliferum* undergoes chemotaxis in response to some amino acids by modifying the “twitch frequency” of swimming (Daniels et al., 1980; Daniels and Longland, 1984). However genes of a bacterial TCS have not been identified in the genome of *S. melliferum* (Lo et al., 2013) or any other *Spiroplasma* species.

*Spiroplasma eriocheiris* (Wang et al., 2011) has been isolated from Chinese mitten crab, *Eriocheir sinensis*, although other species have been isolated from insects or plants. *S. eriocheiris*, is a novel pathogen first found in crustaceans causing mass mortality of *E. sinensis*, as well as shrimps and prawns. In addition, it has had disastrous effects on aquaculture in China in recent years (Xiu et al., 2015). Importantly, *S. eriocheiris* exhibits neurotropic characteristics during the infection process. *S. eriocheiris* infects the central and surrounding nerve systems of crustacean animals (Wang et al., 2004b), including the thoracic ganglion and myoneural junctions, leading to paroxysmal tremor of the pereiopods, and eventually to tremor disease (Wang et al., 2011). It can also infect suckling mice and embryonated chickens, again indicating it has neurotropic characteristics (Wang et al., 2003). *S. eriocheiris* grows faster than other *Spiroplasma* species, and swims actively, which make it an optimal model with which to study the swimming mechanism.

In the present study, we sequenced the genome of *S. eriocheiris*, show novel chemotactic behavior unrelated to conventional TCS, found a novel internal structure of the tip by electron microscopy (EM), and identified the component proteins of the internal architecture. The possibilities for the swimming mechanism are discussed based on the experimental data.

## Materials and methods

### Bacterial strains and growth conditions

The type strain, TDA-040725-5^T^ (= CCTCC M 207170^T^ = DSM 21848^T^) of *S. eriocheiris* (Wang et al., 2011) was cultured under aerobic conditions at 30°C to a late stage of exponential phase. To compare the effects of growth conditions on chemotactic activity, cells were grown in a tissue culture flask or in an anaerobic bag (AnaeroPouch kit provided by Mitsubishi gas chemical, Tokyo, Japan), for aerobic and anaerobic conditions, respectively.

### Genome sequencing

The genome sequence of *S. eriocheiris* was completed by high-throughput sequencing via pyrosequencing. A total of 218,524 sequences with an average length of 246 bp were obtained for the *S. eriocheiris* genome, accounting for 39-fold genome coverages. The sequences were assembled using Newbler software of the 454 suite package, producing 14 contigs ranging from 1.2 to 406 kb. Multiplex PCR was performed to determine the relationship of contigs, and closure of the gaps was performed by sequencing the PCR products. Phred, Phrap, and Consed software package (http://www.phrap.org/phredphrapconsed.html) was used for final assembly and edits. Regions with poor sequencing quality and homopolymers were resequenced. The final consensus accuracy was 99.9982% for the *S. eriocheiris* genome.

### Gene prediction and annotation

Putative protein coding sequences (CDSs) were identified by Glimmer3 (Delcher et al., 1999) and ZCURVE 1.0 (Guo et al., 2003). Functional annotation of CDSs was performed through BLASTP searches against GenBank's non-redundant (nr) protein database, followed by manual inspection. Transfer RNA genes were predicted by tRNAScan-SE (v1.23) (Lowe and Eddy, 1997). The metabolic pathways were constructed using KEGG database (Kanehisa et al., 2004). Lipoprotein was predicted by LipoP 1.0 (Juncker et al., 2003) and searching PROSITE motif PS51257. Protein domain prediction and COG assignment were performed by RPS-BLAST using NCBI CDD library. All against all BLASTP was applied to determine the paralogs. Two sequences in a pair are paralogs if the remaining High-scoring Segment Pairs (HSPs) cover at least 80% of length of the shorter protein and if the identity is greater or equal to 50%.

The TCSs were predicted using both homology search and domain analysis. The homology search was performed against the Prokaryotic 2-Component System (P2CS) database (Barakat et al., 2011) through BlastP with the *E* < 1E-5. The computational domain analysis was performed using the method described in Song et al. (2012). Briefly, all the histidine kinases (HKs) related domains (PF00512, PF07568, PF07730, PF07536, PF06580, PF01627, PF02895, PF05384, and PF10090) and the receiver domain (PF00072) of response regulators (RRs) were used to recognize putative proteins of TCSs.

### Comparative genomics

Unique genes were verified by a TBLASTN search using protein sequences of each strain against the other species' genome sequence. Concatenated protein sequences of 183 orthologous proteins of *Spiroplasma* species were first aligned using MUSCLE (Edgar, 2004), then the conserved alignment blocks were extracted by the Gblocks program (Guindon and Gascuel, 2003). The phylogenetic tree was built using the maximum likelihood method implemented in PHYML (Guindon and Gascuel, 2003).

### Nucleotide sequence accession number

The complete genome sequence and annotations of *S. eriocheiris* were deposited in GenBank under accession numbers CP001973.

### Chemotaxis assay

Cultured cells were centrifuged at 11,000 × g, for 10 min at room temperature (RT) and the pellet was resuspended with a PSF buffer (75 mM sodium phosphate, containing 5% (w/v) sorbitol and 0.1% (w/v) fructose (pH 7.3) containing 0.6% methylcellulose (MC) to the original cell density. For the capillary assay, tips of a mechanical pipet were prepared containing 10 μl of 1% agar with various chemicals to be tested. The tips were soaked in 100 μl cell suspension for 30 min at 30°C. The relative cell number in the tips was estimated by ATP concentration measured by an ATP bioluminescence assay kit HS II (Sigma Aldrich, St. Louis, MO USA).

An agar drop assay was carried out in a chamber, which was assembled by one glass slide, one coverslip and two strips of double-sided tape (Islam et al., 2014). A drop of 1% agar (7 μl) was placed at the center of a glass slide, and a coverslip was attached to the glass slide using double-sided tape. Various possible attractants were selected (Daniels et al., 1980; Daniels and Longland, 1984) and added to the agar drop as necessary. Cell suspension containing 0.6% MC was injected into the chamber. The chamber was sealed by nail polish, and kept for 40 min at RT. The cell distribution at a distance in 4 mm from the agar drop was video recorded with phase-contrast microscopy (BX50, Olympus, Tokyo, Japan). The videos were analyzed by Image J ver.1.37v (http://rsb.info.nih.gov/ij/) as previously reported (Hiratsuka et al., 2005; Nakane and Miyata, 2009, 2012). The cell numbers in a video field 45.5 μm wide and 60.9 μm long were counted manually and averaged for three fields.

For tethered cell experiments, cultured cells suspended in PBS (75 mM sodium phosphate (pH 7.3) and 68 mM NaCl) containing 0.1% fructose adjusted to be equivalent to the original cell density were injected into a flow chamber and observed by phase-contrast microscopy. The liquid in the chamber was replaced by PBS containing 20 μM methionine. Cells attached to glass were video recorded and analyzed.

### Electron microscopy

The cultured cells suspended in PBS to the original density were placed onto a carbon-coated EM grid, and incubated for 10 min at RT. The solution on the EM grid was removed and stained by ammonium molybdate for 1 min. To observe the internal structure, spiroplasma cells were suspended in PBS at 10-fold density of the culture, put on a grid, and incubated for 10 min at RT. The medium was replaced by 3 μl Triton X-100 solution (0.05–0.3% Triton X-100, 1 mg/ml DNase, and 5 mM MgCl_2_ in PBS) and incubated at RT for 30 s. Triton X-100 solution was removed, and the grid was stained for 1 min with 1% ammonium molybdate (vol/vol) and air-dried. The samples were observed by a transmission EM (JEM1010, JEOL, Akishima, Japan) at 80 kV. EM images were captured by a FastScan-F214 (T) charge-coupled-device (CCD) camera (TVIPS, Gauting, Germany). For the isolated filament structure, a 6 μl suspension derived from 10 ml culture was applied to an EM grid. Filaments were picked by e2helixboxer.py (http://blake.bcm.edu/emanwiki/EMAN2/Programs/e2helixboxer). Image averaging was done by EMAN2, version 2.1 (http://blake.bcm.edu/EMAN2/).

### Protein identification

Spiroplasma cells were collected, and washed three times with PBS/G (PBS containing 10 mM glucose), and suspended in PBS, and then 0.05–0.8% Triton X-100, 1 mg/ml DNase, and 5 mM MgCl_2_ were added as the final concentrations. After 20 min incubation on ice, the Triton X-100-insoluble fraction was collected by centrifugation at 19,000 × g for 40 min at 4°C and suspended in PBS. The suspension was analyzed by SDS-PAGE and peptide mass fingerprinting (PMF) using autoflex speed (Bruker Daltonics, Billerica, MA, USA) as reported previously (Nakane et al., 2015).

## Results

### Genes related to swimming in the *S. eriocheiris* genome

**Figure 1:**
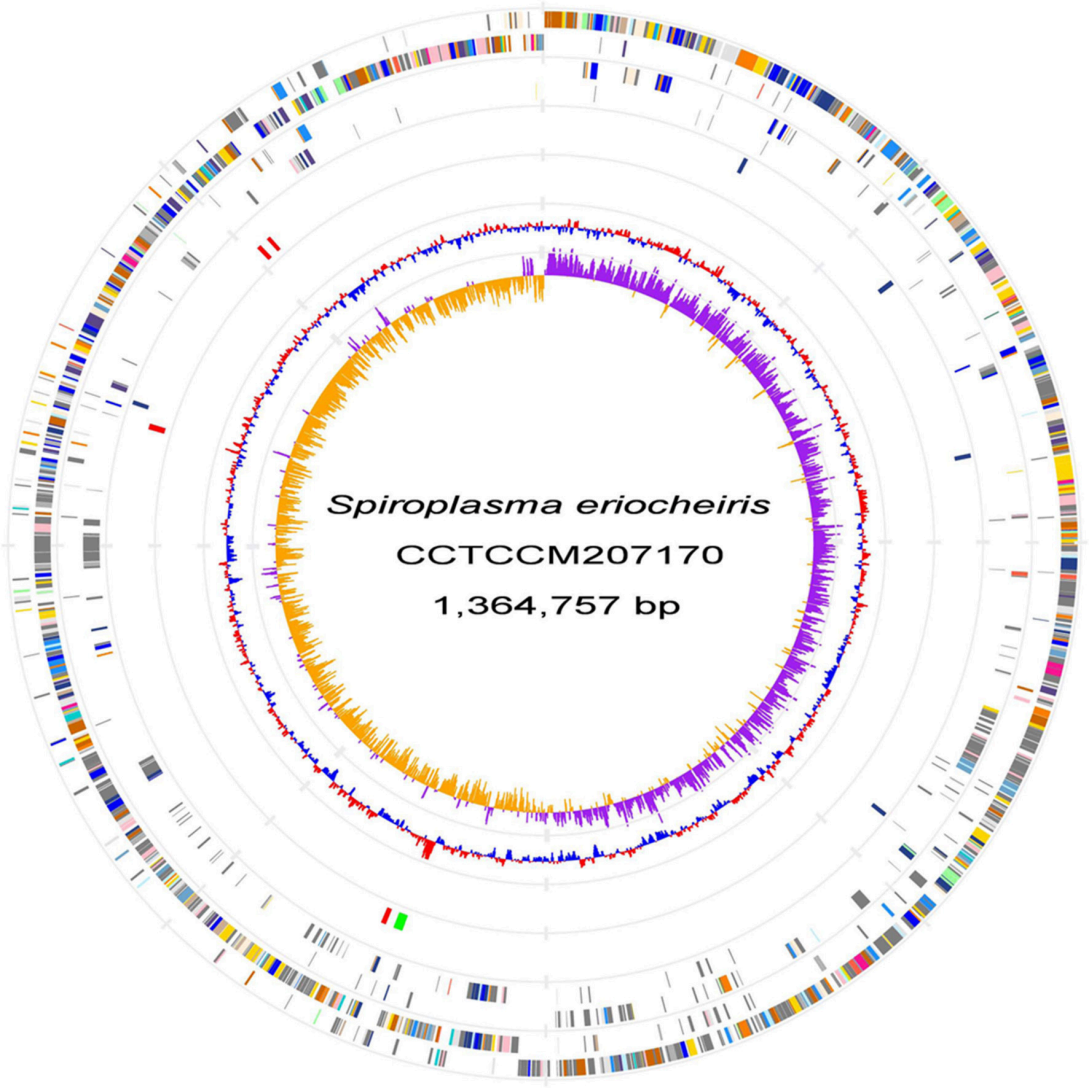
***S. eriocheiris*** **genome**. Chromosome atlas of *S. eriocheiris*. Each concentric circle represents different genomic features. The outermost circle shows predicted protein-coding sequences on both strands, colored for functional categories according to the COG classification. The second circle shows genes specific for the *S. eriocheiris* genome. The third circle shows tRNA genes on plus (blue) and minus (red) strands, and ribosomal RNA genes (green). The forth circle shows IS elements. The fifth circle shows the mean centered G + C content using a 1 kb window colored red and blue for values above and below, respectively. The innermost circle shows GC skew (G−C)/(G+C) calculated using a 1 kb window colored purple and yellow for values plus and minus, respectively.

We sequenced the genome of *S. eriocheiris*, a single, circular DNA of 1,364,757 bp (Figure 1). *S. eriocheiris* showed different genomic characters from the other 13 *Spiroplasma* species, harboring the second largest genome in the *Spiroplasma* genus, only 100 kb smaller than corn parasite *S. kunkelii*, and at least 100 kb larger than other *Spiroplasma* species (Table S1). The genome similarity analysis by GGDC (Meier-Kolthoff et al., 2013) of the *Spiroplasma* genomes also indicates the high divergence between *S. eriocheiris* and other *Spiroplasma* species, with 70% similarity to only *S. mirum* (Table S2).

The cytoskeletal proteins, Fibril (SPE_0666) (Williamson et al., 1991), five MreB proteins (Ku et al., 2014) (SPE_0470, SPE_1224, SPE_1228, SPE_1230, and SPE_1231) and FtsA (SPE_0753)—which are related to eukaryotic actin—and FtsZ (SPE_0752)—related to eukaryotic tubulin—were found.

To date, all bacterial species that undergo chemotaxis are known to have genes classified as TCSs. To identify proteins related to the conventional TCS in the genome, a homology search against all the “Prokaryotic 2-Component System” (P2CS) database (Barakat et al., 2011) was firstly performed. However, no TCS proteins were found in the *S. eriocheiris* genome. We then analyzed all of the domains encoded by the genome, and failed to find any known domains of histidine kinases (HKs), response regulators (RRs), and other protein components that are involved in well-studied chemotaxis systems, like Methyl-accepting chemotaxis protein (MCP) signaling domain.

### Chemotaxis examined by capillary assay

**Figure 2:**
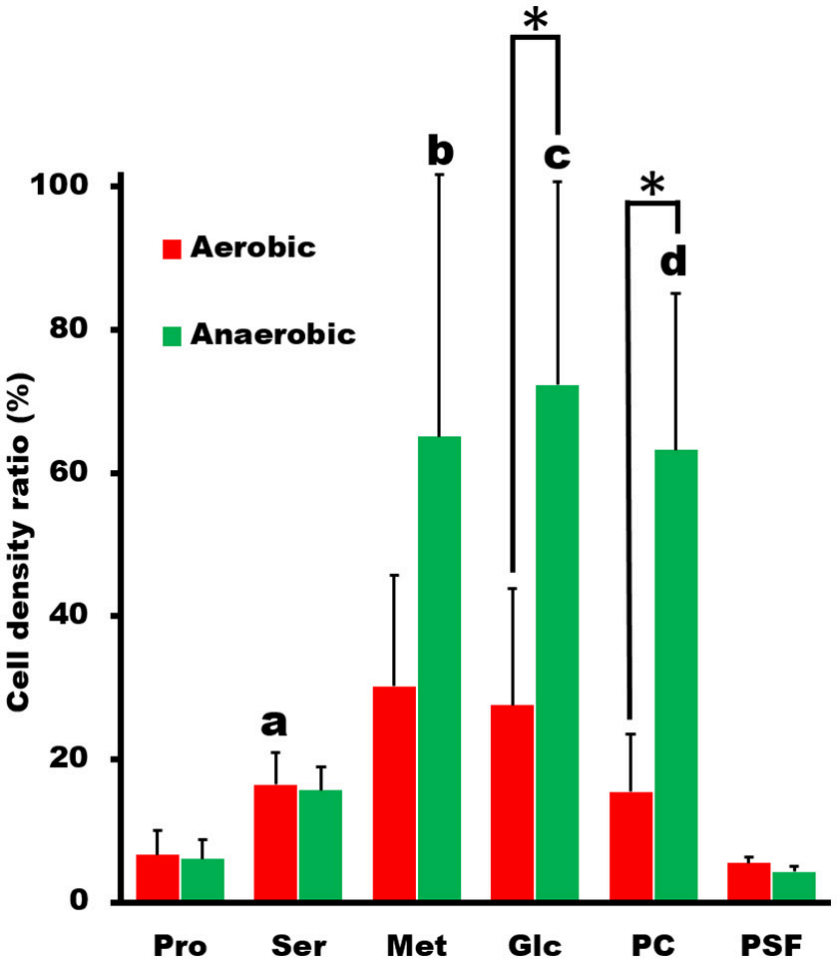
**Capillary assay to examine chemotaxis of *S. eriocheiris***. The capillaries containing 10 mM proline (Pro), 10 mM serine (Ser), 10 mM methionine (Met), 20 mM glucose (Glc), and 5 mg/ml phosphatidylcholine (PC) in PSF were soaked into a cell suspension for 30 min. The cell densities in the capillary relative to that in the cell suspension examined were estimated by ATP concentration. The values from three experiments were averaged. The conditions for cultivation are represented by colors. The statistical significance was determined by Student's *t*-test (*p* < 0.05) between the aerobic and anaerobic conditions marked with an asterisk (^*^), and between the chemicals and PSF marked with characters, a, b, c, and d at the top of the bar.

Next, we examined the chemotactic ability of *S. eriocheiris* cells by using a capillary assay. The cells cultured in either aerobic or anaerobic conditions were suspended in a 100 μl PSF buffer containing 0.6% MC in a test tube. Pipet tips filled with various attractants, which were reported as an attractant for other microorganisms (Daniels et al., 1980; Daniels and Longland, 1984), were placed into the buffer. After incubation for 30 min at 30°C, the contents of tips were recovered and titrated for ATP concentration, which is known to be linearly related to the cell number (Dexter et al., 2003). ATP concentration is useful as a maker of cell number for *Mollicute* species which are not suitable for counting CFU (colony forming unit) because of their sticky cell property and slow growth rates. The cell numbers estimated from the ATP concentrations were obviously higher in the capillary containing the putative attractants, showing that more spiroplasmas swam into the pipet tips containing these substrates than in the control (Figure 2). It is unlikely that they propagated in the capillary because they need 150 min for doubling even in the growth medium. Interestingly, the chemotactic activity was more obvious when the spiroplasmas grew under anaerobic conditions vs. aerobic ones. These results, together with the genome analysis, show that *S. eriocheiris* performs chemotaxis dependent on aerobic conditions without using conventional TCS.

### Chemotaxis examined by agar-drop assay

**Figure 3:**
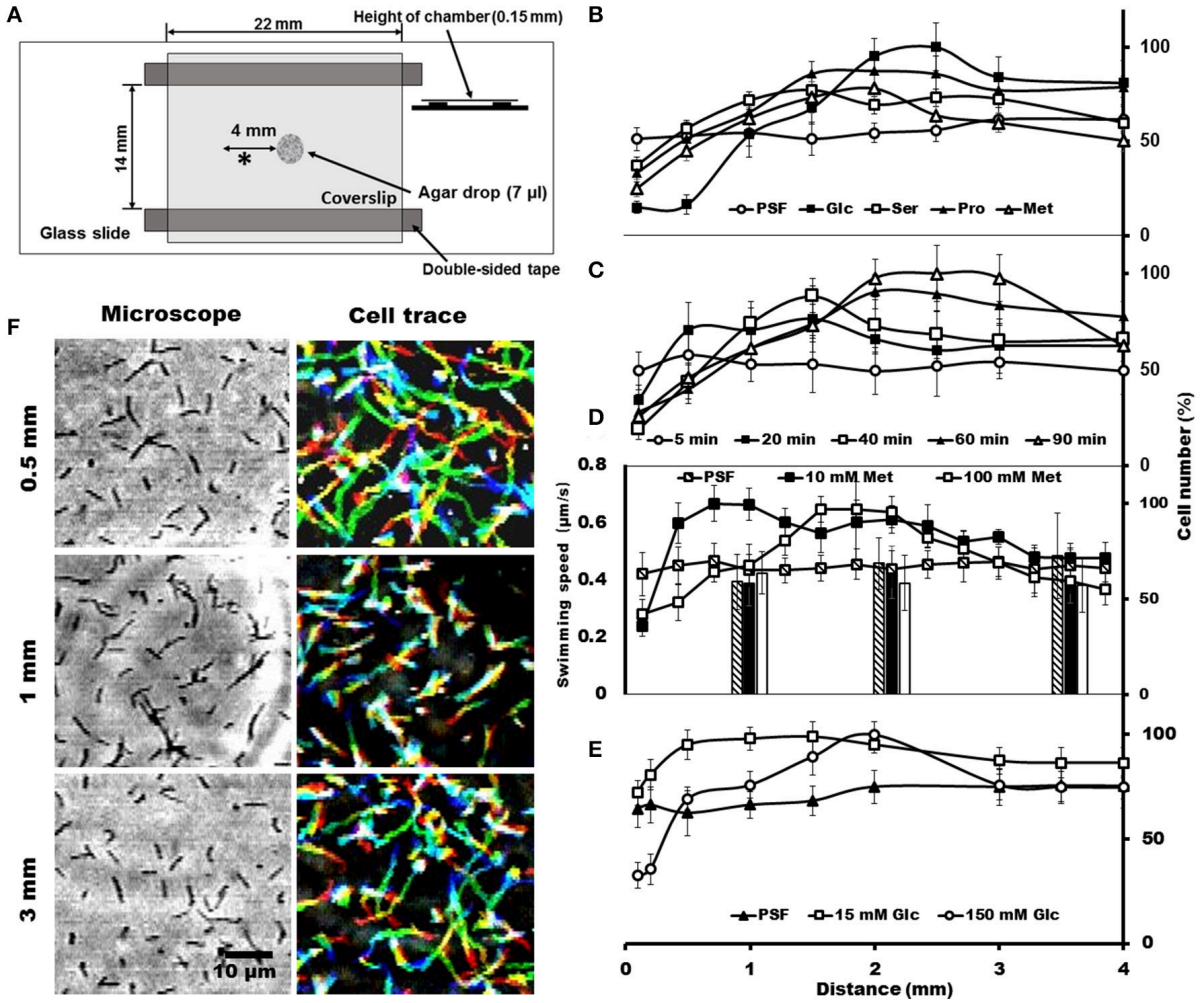
**Cell distributions and behaviors in the agar-drop assay. (A)** Schematic representation of the chamber. A drop of 1% agar (7 μl) was placed at the center of a glass slide, and a coverslip was attached to the glass slide using two pieces of double-sided tape. Various chemicals were added to the agar drop as necessary. The observing area is marked by an asterisk (^*^). **(B)** Cell numbers at different positions from the agar drop containing either 200 mM glucose, 150 mM serine, 150 mM proline, or 100 mM methionine. An agar drop without an attractant was used as a control. The number of cells is shown by the ratio relative to the highest point. The maximum cell numbers ranged from 425 to 770 through panels **(B–E)**. **(C)** Cell numbers counted at different positions from the agar drop containing 100 mM methionine after the following different incubation times: 5, 20, 40, 60, and 90 min. **(D)** Cell numbers at different positions from the agar drop counted in the presence of 0, 10, and 100 mM methionine. The bars indicate the swimming speeds of cells at different positions from the agar drop. **(E)** Cell numbers at different positions from the agar drop were counted in the presence of 0, 15, and 150 mM glucose. **(F)** Phase-contrast (left) and cell-trace (right) images exhibiting the cell behaviors at different positions 0.5, 1, and 3 mm from the agar drop containing 100 mM methionine. Cell trajectories for 8 s are presented as a stack, changing their color from red to blue.

To trace the cell behaviors responding to the attractants, an agar-drop assay was used, in which the density and movements of cells around an agar drop containing the attractant were traced by optical microscopy (Islam et al., 2014). The solutions containing 1% agar were placed at the center of a chamber that was assembled using a glass slide, coverslip, and double-sided tapes (Figure 3A). Then, the remaining space of the chamber was filled with the cells suspended in PSF containing 0.6% MC. After 40 min at RT, the cell images were video recorded, and the numbers and the behaviors of cells were analyzed at various positions from the agar drop. We examined the same set of chemicals with those examined in the capillary assay and found that the cells distributed differently depending on the chemicals, although they distributed evenly throughout when the drop contained only PSF (Figure 3B). In the presence of attractants, the cell density at the position immediately adjacent to the agar drop was lower than that without attractant and increased with distance from the agar drop, reaching a peak at a point that may represent an optimal concentration in the diffusion gradient of the attractant. The peak positions were around 1.5, 1.5, 2.2, and 2.5 mm, respectively, for 150 mM serine, 150 mM proline, 100 mM methionine, and 200 mM glucose, suggesting that the cells came to the optimal concentration for each attractant (Figure 3B). These results are not inconsistent with the above results that the cells moved into the capillary filled by agar (Figure 2), because higher concentrations of chemicals were applied to the agar drop assay. Next, we examined the time dependency of the cell distribution (Figure 3C). The cell density at different positions from the agar drop containing 100 mM methionine was examined after various times. The cells distributed evenly at time 0, and then formed a density peak with time, and the peak moved. This observation shows that the cells moved synchronously with the diffusion of methionine, by tracing the most optimal concentration as it diffused outward from the agar drop. Then, we examined the cell density at various positions, with 10 and 100 mM methionine, the most effective attractant at 40 min (Figure 3D). The cells distributed with a density peak at 0.3 and 1.1 mm in the presence of 10 and 100 mM methionine, respectively, showing that the cells moved to the zones of the optimal concentration of methionine. A similar result was obtained also for glucose (Figure 3E). Next, we traced the swimming of individual cells at three positions, 0.5, 1.0, and 3.0 mm, from the agar drop containing 100 mM methionine (Figure 3F). The cells showed longer trajectories in the low-density area than in the high-density area. These results suggest that *S. eriocheiris* cells can sense attractive chemicals and swim to the most favorable concentration by changing their swimming behaviors.

### Swimming behaviors

**Figure 4:**
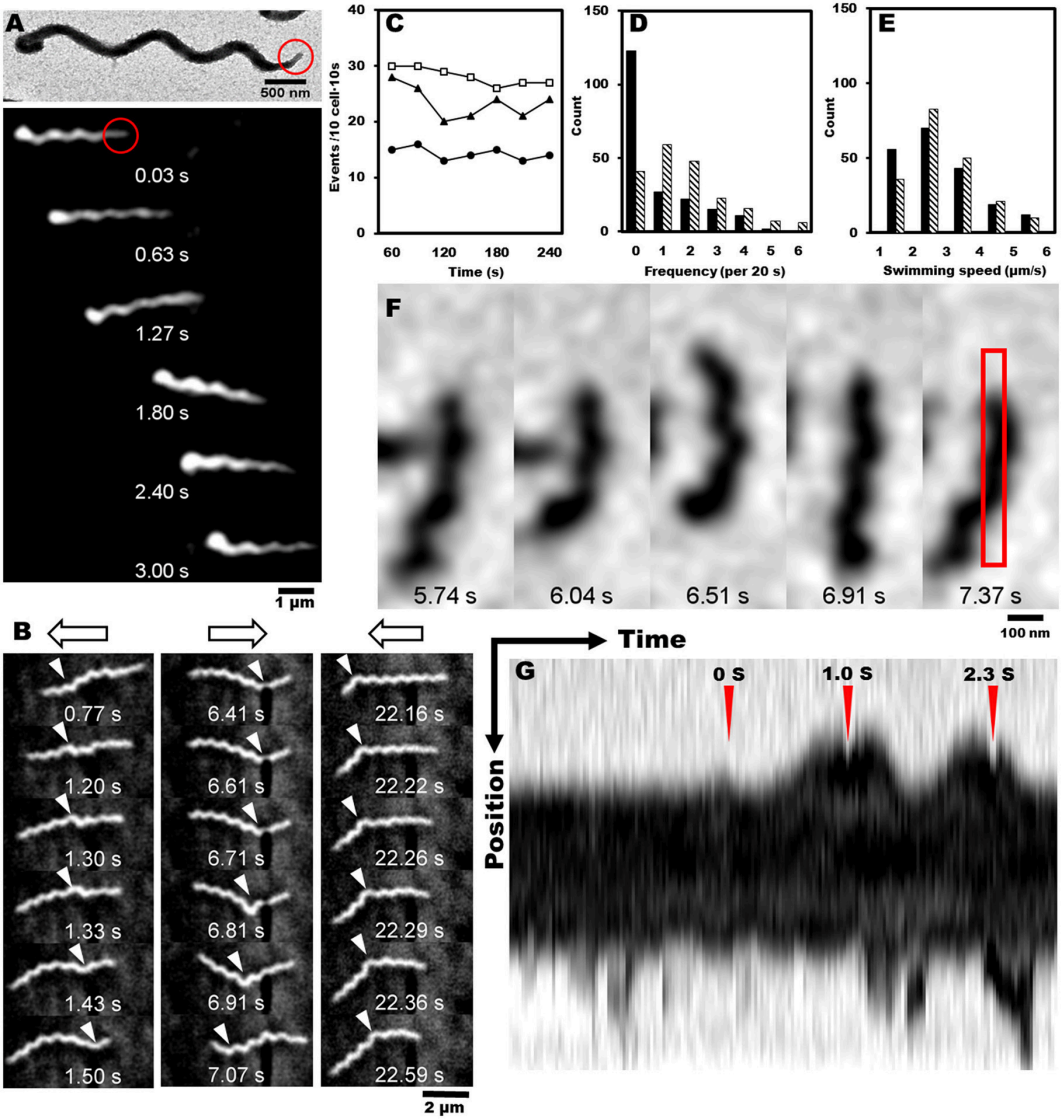
**Reversal behaviors of swimming and tethered cells. (A)** Upper: negatively stained EM image of spiroplasma cell, the tip structure of which is marked by a red circle. Lower: montage image showing that spiroplasma swim in the direction of the tip structure. **(B)** Consecutive cell images from a video showing reversal behavior of swimming. Open arrows on the top indicate swimming directions, and white triangles indicate kink positions. **(C)** Reversal frequencies of ten cells in 10 s in the presence of various concentrations of methionine, 0 (solid circle), 3 (solid triangle), and 30 mM (open square). **(D)** Reversal frequencies of swimming cells from aerobic (solid) and anaerobic (hatch) cultivations. **(E)** Swimming speed of cells from aerobic (solid) and anaerobic (hatch) cultivations. **(F)** Consecutive images of a cell attached to the glass slide. The area for kymograph is boxed in the rightmost image. **(G)** Kymograph of cell image after the cell was exposed to 20 μM of methionine at time 0 marked by red triangle. Reversal behaviors were observed around the peak times at 1.0 and 2.3 s marked by red triangles.

Most motile bacteria have cell polarity and exhibit chemotactic activity by switching moving directions (Adler, 1966; Porter et al., 2011; Typas and Sourjik, 2015). Generally, flagellated bacteria sense the change in concentrations of attractants and repellents with time, and control the reversal frequency between counterclockwise (CCW) and clockwise (CW) in flagella rotation. Interestingly, *S. eriocheiris* cells have polarity defined as a tapered end, also called tip, which is observed in a few species (Garnier et al., 1981; Ammar et al., 2004; Wang et al., 2004a). Cells mostly swam in the direction of the tip (Figure 4A, Movies 1, 2). For example, 30 in 35 swimming cells moved in the direction of the tip in 1 s. In addition, the cells changed swimming directions frequently (Figure 4B and Movie 3). The difference in the trajectory lengths among different positions in the agar drop assay likely resulted from the reversal frequency of the swimming direction, because the swimming speeds measured every 1 s did not change significantly by the cell position in the chamber (Figure 3D). To examine this assumption, we counted the reversal events of cells in different conditions. The frequency of reversals clearly depended on the methionine concentration as 13, 20, and 29 reversal events were observed for 0, 3, and 30 mM of methionine, respectively. The cells did not show any adaptive response in 4 min, namely the reversal frequency did not change significantly (Figure 4C). The frequency depended on the aerobic condition for cultivation (Figures 4D,E, Movies 1, 4), consistent with the results of the capillary assay shown in Figure 2.

Next, we tried a tethered cell assay, in which the behavior of a single cell fixed on a solid surface was analyzed. We inserted the cells suspended in PSF into a tunnel slide, and found a cell occasionally attached to the glass surface (Figure 4F). We replaced the PSF in the tunnel slide with fresh PSF containing 20 μM methionine, started to record the cell images as soon as possible, and analyzed the reversal behaviors. When the cells were exposed to methionine, they switched their behaviors from one to both directions, demonstrating reversal behavior (Figure 4F and Movie 5). The kymograph of a cell image shows two reversals at 1.0 and 2.3 s after the cell was exposed to methionine at time 0 (Figure 4G).

### Internal structures visualized by EM

**Figure 5:**
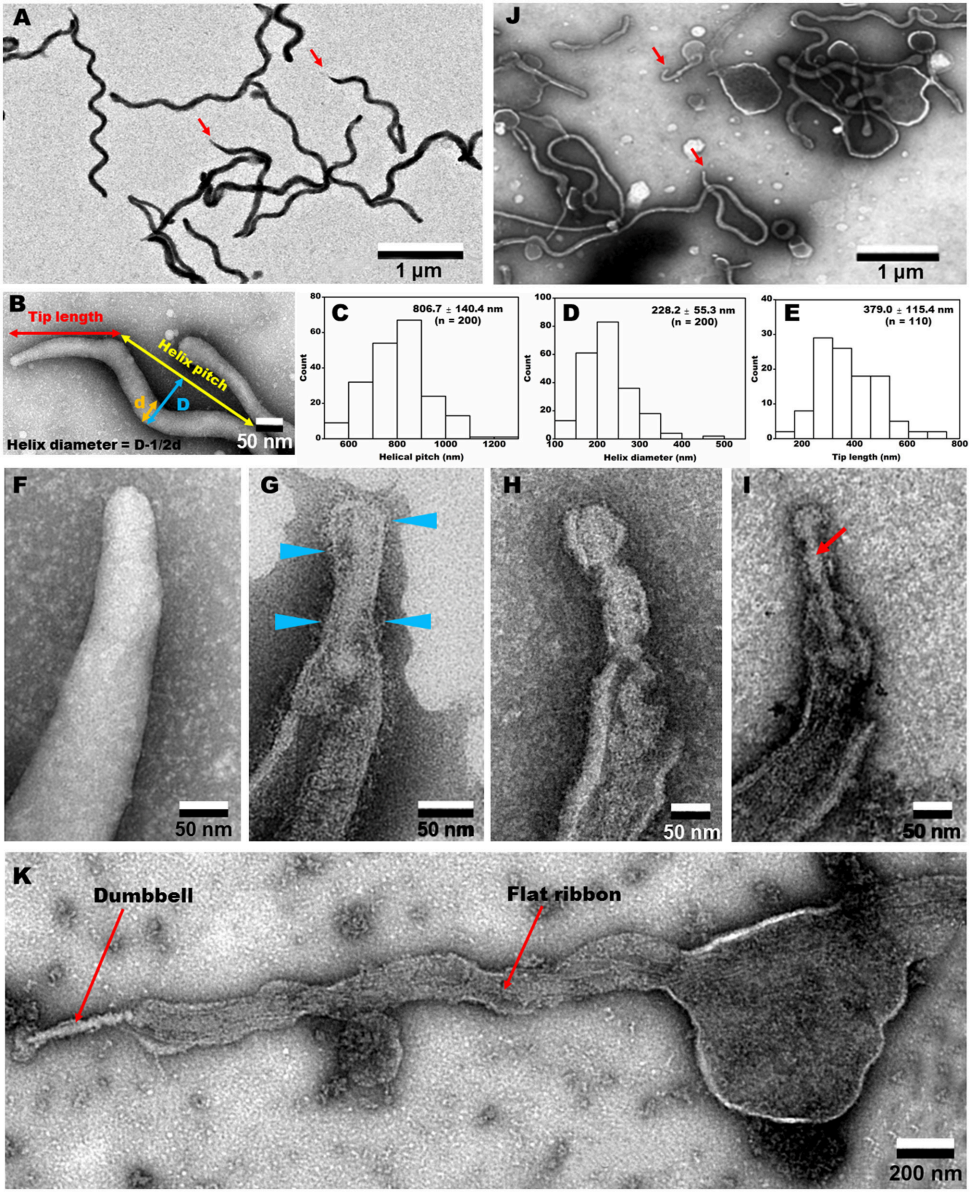
**Negative-staining EM images of cells treated with different concentrations of Triton X-100. (A)** Field image of intact cells. Red arrows indicate the tip structure. **(B)** Magnified cell image and definition of dimensions. The pitch **(C)** and diameter **(D)** of helix were measured. **(E)** Tip length distribution. **(F–I)** Tip structure treated with 0 **(F)**, 0.05 **(G)**, 0.1 **(H)**, and 0.3% **(I)** Triton X-100. Blue triangles indicate filamentous structures on the surface. A dumbbell-shaped structure is marked by a red arrow in **(I)**. **(J)** Field image of cells treated with 0.1% Triton X-100. Red arrows indicate the tip structures. **(K)** Whole image of a cell extracted by 0.3% Triton X-100. A dumbbell-like structure is connected by a flat ribbon.

The EM images of negatively-stained intact cells revealed helical cell shapes (Figure 5A) with dimensions 2.0 to 10.0 μm long, 77.6 ± 8.8 nm wide, 806.7 ± 140.4 nm helix pitch, and 228.2 ± 55.3 nm helix diameter (*n* = 200) (Figures 5B–D). A pole of an intact cell was featured with a tapered tip structure (Figures 5A,B), as observed by optical microscopy (Figure 4A). The length of an intact tip structure distributed around 379.0 ± 115.4 nm (*n* = 110; Figure 5E).

**Figure 6:**
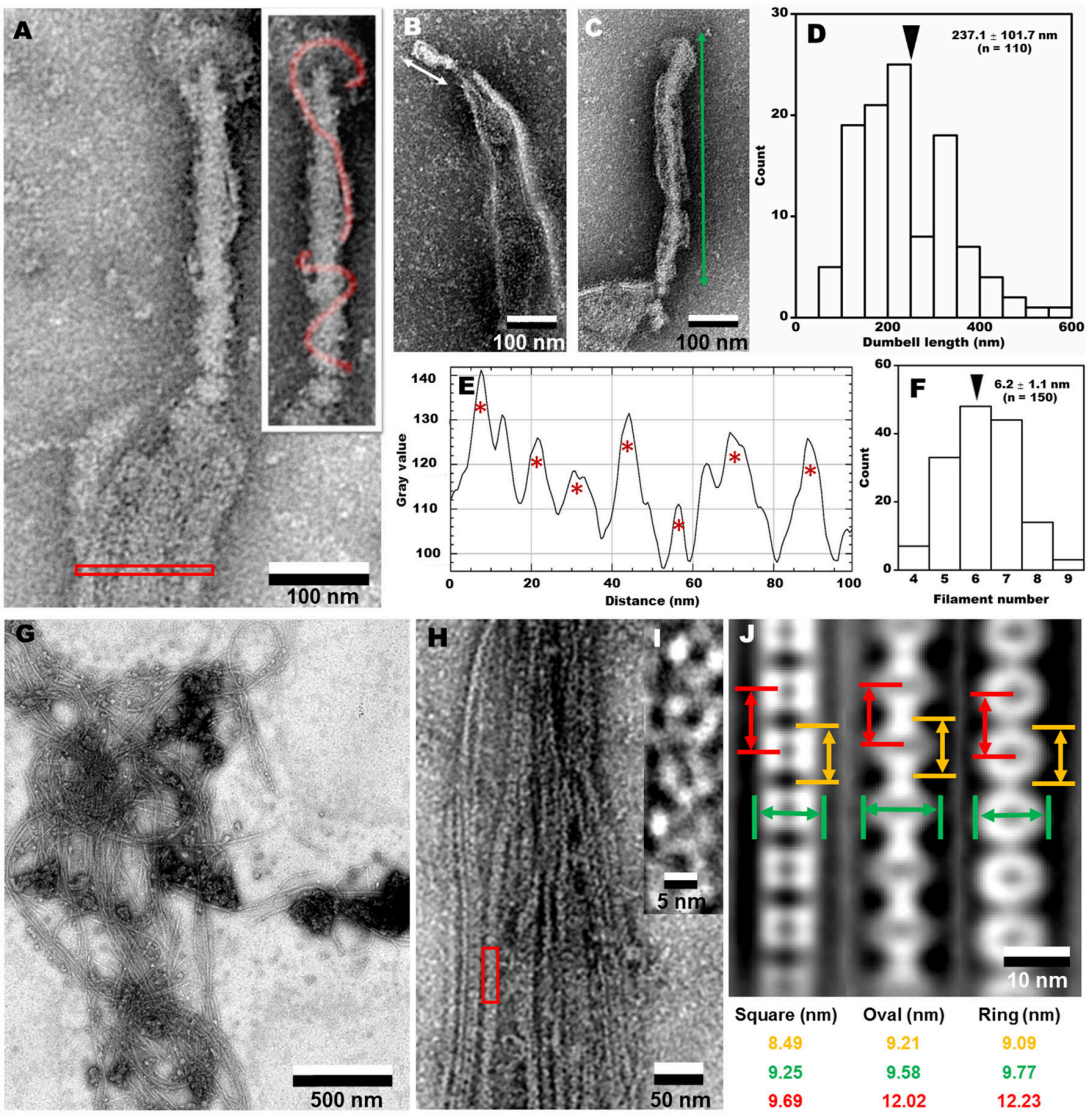
**Analyses of internal structures by negative-staining EM. (A)** A high resolution image of tip structure shown in Figure 5K. The helical structure around the dumbbell is colored in the inset and the area for ribbon profile is boxed. **(B,C)** Short **(B)** and long **(C)** dumbbells exposed by Triton X-100. **(D)** The length distribution of dumbbell structure. The average is marked by a black triangle. **(E)** The density profile of ribbon in the cell indicated by a red box in **(A)**. Seven filaments were detected as peaks marked by red asterisks. The peaks were detected only when the peak was higher than 100 units compared to the adjacent lower inflection points. The filament numbers in a cell are presented in **(F)**. The average number is marked by a black triangle. **(G)** Field image of isolated filaments after the treatment with 0.8% Triton X-100. **(H)** Magnified images of isolated filaments. **(I)** The red box area in **(H)** was magnified. **(J)** Three types of averaged filaments, with type names and dimensions in the bottom.

To clarify the architecture of the internal structure of *S. eriocheiris*, we observed the cell after treatments with various concentrations of Triton X-100 (Figures 5F–J). After treatment with 0.05% Triton X-100, some filamentous structures were observed on the surface and in the cell (Figure 5G). Next, we treated the cells with 0.1% (Figures 5H,J) and 0.3% (Figure 5I) Triton X-100. After treatment with 0.3% Triton X-100, the major parts of cell membrane were removed, and the intracellular structures were exposed (Figures 5I,K). We observed that the internal structure of a spiroplasma cell is composed of a dumbbell-shaped structure and a flat ribbon-like structure. The dumbbell-shaped structure at the tip was connected by a flat ribbon that traces a short line in the helical cell from the tip to the other pole (Figure 5K). Interestingly, one spiral filament was found around the dumbbell structure (Figure 6A). The length of the dumbbell was approximately 237.1 ± 101.7 nm (*n* = 110; Figures 6B–D). The ribbons were composed of five to eight filaments (Figures 6E,F), consistent with a previous study of *S. melliferum* (Kürner et al., 2005; Cohen-Krausz et al., 2011). Next, to obtain clearer images of the filaments, we treated the cells with 0.8% Triton X-100, recovered the insoluble fraction by centrifugation at 19,000 × g for 40 min, and observed the fraction by EM (Figures 6G–I). The filamentous structures were clearly visible as connected ring units. Image averaging showed that the filaments can be classified into three structures, namely square, oval and ring, averaged for 753, 1045, and 857 particles, respectively (Figure 6J). The dimensions were 8.49 long, 9.25 nm wide with 9.69 nm intervals for square; 9.21 long, 9.58 nm wide with 12.02 nm intervals for oval; and 9.09 long, 9.77 nm wide with 12.23 nm intervals for ring. The oval and the ring are consistent with previous studies of *S. melliferum* (Cohen-Krausz et al., 2011) but the square was only identified in this study. More experimental information is needed to clarify the relationship among these structures.

### Identification of protein components of the internal structure

**Figure 7:**
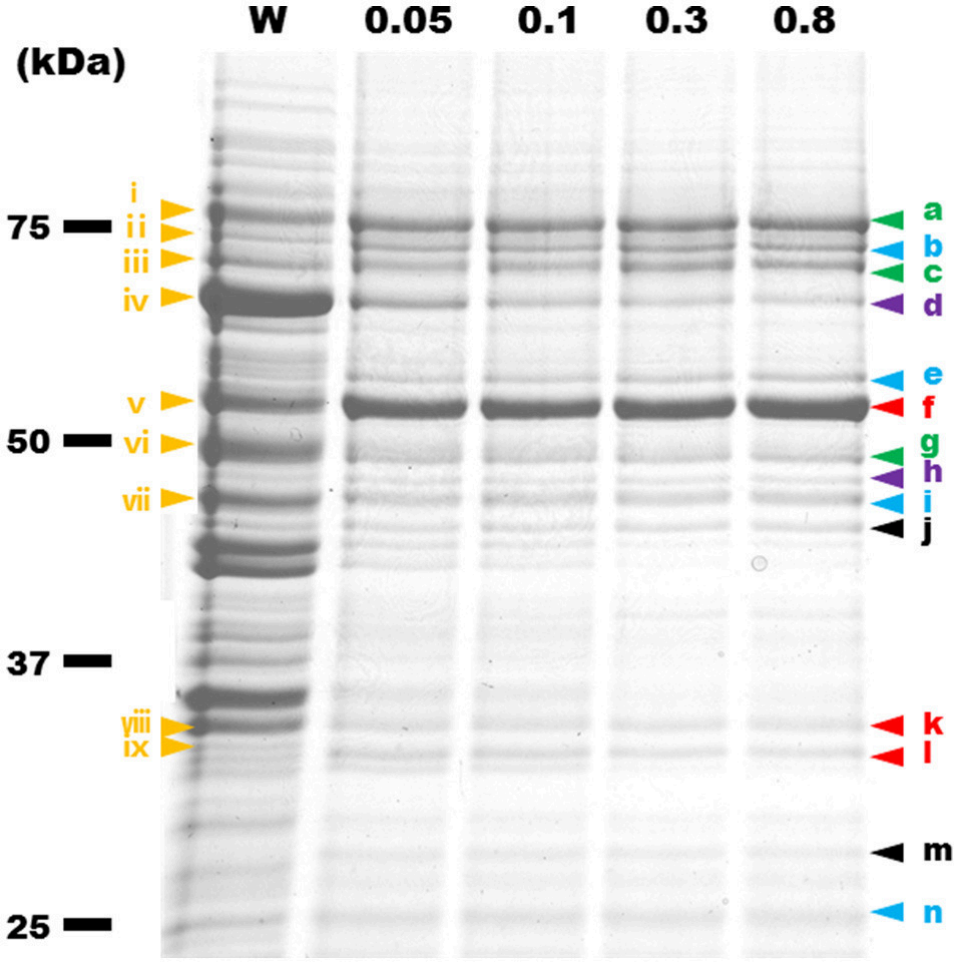
**Protein profiles of Triton X-100-insoluble fractions**. The fractions were subjected to SDS-PAGE of 10% polyacrylamide and stained by Coomassie Brilliant Blue (CBB). The amount of fraction applied to each lane was adjusted to the same number of cells. W, the whole-cell lysate. Insoluble fractions after treatment by 0.05, 0.1, 0.3, and 0.8% Triton X-100 are marked by the Triton X-100 concentrations. Yellow triangles with a Roman numeral i to ix indicate protein bands that were identified by MALDI-TOF mass spectrometry. The results are summarized in Table S3. The protein bands identified from the fractions treated by 0.8% Triton X-100 are marked by a triangle indicated a to n. Green, blue, purple, red, and black colors show the features of proteins, respectively, transmembrane, lipoprotein, ATPase activity, filamentous, and other.

To identify the protein components of the internal structures, the cells were treated with various concentrations of Triton X-100. The insoluble fractions were collected and analyzed by SDS-PAGE (Figure 7). Fewer proteins remained in the insoluble fractions after the treatments with higher concentrations of Triton X-100. The 14 protein bands in the insoluble fraction after 0.8% Triton X-100 treatment were excised, and subjected to protein identification by MALDI-TOF mass spectrometry. Sixteen proteins were identified (Table 1), including Fibril protein (SPE-0666) (Townsend et al., 1980; Williamson et al., 1991) and four MreB proteins (SPE-0470, SPE-1224, SPE-1230, and SPE-1231) (Ku et al., 2014). The band positioning between m and n was not identified because of weak mass spectrometry signals, which may be caused by the mixture of source proteins. Nine protein bands through i to ix from the whole cell lysate at the corresponding positions with those in the insoluble fractions were also analyzed by mass spectrometry (Table S3). DnaK (SPE-0178) detected as band d might be a contamination because it was abundant in the whole cell fraction. The other 15 proteins should be involved in the filaments observed by EM as the components. The identified proteins were classified into five categories, i.e., (I) probable subunits of filaments including Fibril and MreB proteins, colored red in Figure 7 and Table 1; (II) proteins containing transmembrane segments, colored green; (III) lipoproteins, colored blue; (IV) proteins predicted for ATPase activity, colored purple; and (V) other, colored black.

**Table 1:** **Identification of protein bands shown in Figure 7**.

**Protein band**	**Gene ID**	**Annotation**	**Amino acid**	MW^*^ kDa
a	SPE-1201	Conserved hypothetical protein	769	85.8
b	SPE-1165	Lipoprotein	721	80.5
c	SPE-0013	FtsH	692	77.0
d	SPE-0178	DnaK	600	65.9
e	SPE-1166	Lipoprotein	571	64.4
f	SPE-0666	Fibril	512	58.6
g	SPE-0723	Hypothetical protein	517	58.4
h	SPE-0121	F_1_ ATP synthase beta subunit	464	50.4
i	SPE-0123	Lipoprotein	499	55.0
j	SPE-0075	ABC-type transport system substrate-binding protein	468	51.4
k	SPE-1231	MreB5	350	38.5
	SPE-0470	MreB1	346	37.9
l	SPE-1224	MreB2	348	37.9
	SPE-1230	MreB4	369	40.7
m	SPE-0453	1-acyl-sn-glycerol-3-phosphate acyltransferase	265	31.2
n	SPE-0708	Lipoprotein	221	25.1

Green, blue, purple, red, and black colors show the features of proteins transmembrane, lipoprotein, ATPase activity, filamentous, and other, respectively. SPE-1201 and SPE-0013, and SPE-0723 have two and one transmembrane segments, respectively. Conserved hypothetical protein is found only in Spiroplasma species. Hypothetical protein is conserved in other bacterial species.

* *Molecular mass predicted from amino acid sequences*.

## Discussion

### Chemotaxis without conventional TCS

The polarized cell morphology and the tip structure have been previously reported for spiroplasmas (Garnier et al., 1981; Ammar et al., 2004). In addition, the relationship of the cell polarity to the cell division cycle has been reported (Garnier et al., 1981), but the relationship between the tip and swimming direction has not been examined. In the present study, we clearly showed that *S. eriocheiris* cells swim in the direction of the tip, at least for the most part (Figure 4A and Movie 2). Considering that spiroplasma swims by the switch of helicity traveling from front to back (Shaevitz et al., 2005; Wada and Netz, 2007, 2009), the tip may play a role in changing the helicity.

Moreover, we showed that the reversal of the moving direction is linked to chemotactic activity (Figure 4). The reversal motion may be common amongst the species since it was previously observed that the chemotaxis of *S. melliferum* is linked to the “twitch frequency” (Daniels et al., 1980; Daniels and Longland, 1984). The chemotaxis to phosphatidylcholine found here in *S. eriocheiris* may be evidence of neurotropic characteristics (Figure 2) (Wang et al., 2011). Interestingly, the chemotaxis of *S. eriocheiris* was more obvious when it was cultivated under anaerobic conditions vs. aerobic ones (Figures 2, 4). This may be the cause of the phenomenon that *S. eriocheiris* is more epidemic in summer when the oxygen concentration in fresh water of their habitat is lower than other seasons (Benson and Krause, 1984; Wang et al., 2004b). The anaerobic conditions may induce the expression of genes essential for the chemotaxis or switch the unknown chemotaxis mechanism in *S. eriocheiris*.

Even though genes for TCS were not found in the genome of spiroplasmas, *S. eriocheiris* behaves in a similar way with respect to chemotaxis as other motile bacteria; the bacteria manage to reach and/or avoid chemicals by changing the reversal frequency of propulsion (Porter et al., 2011; Typas and Sourjik, 2015). These facts can be explained by two possibilities, (i) spiroplasmas also have a TCS distantly related to other motile bacteria, or (ii) they perform chemotaxis by a mechanism different from other motile bacteria.

### Dumbbell and ribbon structures

In the present study, we identified an internal structure in the tip, designated as dumbbell, by Triton X-100 extraction (Figures 5, 6A). Although this structure is reminiscent of the core structure of the *M. pneumoniae* tip (Nakane et al., 2015; Kawamoto et al., 2016), none of the protein encoding genes in the *S. eriocheiris* genome showed sequence similarities to the 15 components of the *M. pneumoniae* tip structure (Figure 7 and Table 1; Nakane et al., 2015; Miyata and Hamaguchi, 2016). Some groups of mycoplasmas are known to bind to sialylated oligosaccharides on solid surfaces through the tip structure (Kasai et al., 2013, 2015). The spiroplasma tip may have a similar role to those of mycoplasmas, because spiroplasmas appear to bind to host cells through the tip in sectioning images (Ammar et al., 2004).

### Components of internal structure

Our results confirmed that Fibril, an abundant internal protein forms the ribbon, consistent with previous studies of *S. melliferum* (Kürner et al., 2005; Trachtenberg et al., 2008). We also showed that at least four of five MreB proteins encoded by the genome were found in the insoluble fraction (Figure 7 and Table 1), consistent with a previous study which identified an MreB protein in an insoluble fraction of *S. melliferum* (Trachtenberg et al., 2008). Fibril and the four MreB proteins may be involved in the ribbon structure. Interestingly, five classes of MreB proteins were found in all *Spiroplasma* genomes sequenced, although most bacteria have only one MreB homolog and *Mollicute* species other than spiroplasmas do not have MreB genes (Table S4), suggesting that the combination between Fibril and MreB proteins is essential for the unique swimming of spiroplasmas (Ku et al., 2014).

The other 11 proteins that remained after 0.8% Triton X-100 treatment may also be components of the internal structure. However, we need additional experiments to conclude this, because the proteins containing transmembrane segments detected as protein bands a, c, and g may remain in the fraction because of their insolubility. The proteins predicted for ATPase activity may be candidates for the motor of spiroplasma swimming, although the direct energy source has not been specified so far.

## Conclusion

It has been suggested that spiroplasmas swim by propagating a change in helicity from front to back (Trachtenberg, 2004; Shaevitz et al., 2005). The original form of a spiroplasma cell is a right-handed helix and the handedness switches after the kinks. As a ribbon structure lines the inside of the helix, it is reasonable to assume that the cell helicity and the kink are driven by the conformational changes in the ribbon. We identified Fibril, as previously reported, as well as four MreB proteins, for the first time, in the fraction enriched for the ribbon. The combination of these proteins may cause the conformational change necessary for swimming. As the change in helicity occurs at the front end, the dumbbell structure may be involved in this switch, possibly through torque generation at the connecting part. This switch sometimes travels to the opposite direction and causes reversal movements. As the reversal occurs responding to chemoattractants, it is likely linked to chemotaxis of spiroplasma. The change in reversal frequency is common with the conventional bacterial chemotaxis governed by a TCS. Interestingly, genes encoding the TCS cannot be found in *Spiroplasma* genomes. Considering the assumption that the change in cell helicity is governed by the tip, the tip may have some role in the chemotactic activities.

## Author contributions

Conception and design were done by PL, NT, DN, and MM (Chemotaxis and inside structures parts), HZ, QM, WG, SW, GZ, and WW (Genome part). Collection and assembly of data were done by PL, NT (Chemotaxis and inside structures parts) and HZ (Genome part). Manuscript writing was done by PL and MM. Final approval of manuscript was done by PL, WW, and MM.

## Funding

This study was supported by China Scholarship Council (CSC) to PL ([2015]3022), National Natural Science Foundation of China (NSFC) to WW (31272686), Project Funded by the Priority Academic Program Development of Jiangsu Higher Education Institutions to WW, Ministry of Education, Culture, Sports, Science, and Technology (MEXT) to MM (24117002), Japan Society for the Promotion of Science (JSPS) to MM (21249030) and Japan Society for the Promotion of Science (JSPS) to MM (24390107).

### Conflict of interest statement

The authors declare that the research was conducted in the absence of any commercial or financial relationships that could be construed as a potential conflict of interest.

## Supplementary material

Movie 1 **Swimming of *S. eriocheiris* cultivated in aerobic conditions**. The cultured cells were observed in the presence of 0.6% MC by dark-field microscopy. The image width is 13.8 μm. Real time.

Movie 2 **Directed cell shape and swimming direction of *S. eriocheiris***. Cells cultured in aerobic conditions were suspended in a PSF buffer containing 0.6% MC and observed. The cells are polarized as determined by a tip structure and swim in the direction of the tip. The image width is 9 μm. Real time.

Movie 3 **Reversal behaviors of *S. eriocheiris* swimming**. Cells cultured in aerobic conditions were suspended in a PSF buffer containing 0.6% MC and observed. The cells changed swimming direction frequently. The image width is 9 μm. Real time.

Movie 4 **Swimming of *S. eriocheiris* cultivated in anaerobic conditions**. The cultured cells were observed in the presence of 0.6% MC by dark-field microscopy. The image width is 13.8 μm. Real time.

Movie 5 **Tethered cell**. Cells cultivated in the aerobic conditions were suspended in PBS (75 mM sodium phosphate (pH 7.3), and 68 mM NaCl) containing 0.1% fructose, and poured into a tunnel slide. A cell stuck to the glass surface was traced. At time 3.7 s in this movie, PBS was replaced by PBS containing 20 μM of methionine. The tethered cell showed reversal behaviors twice. The image width is 390 nm. Real time.

Table S1 **General features of *Spiroplasma* genomes**.

Table S2 **Similarity between *S. eriocheiris* and other *Spiroplasma* genomes calculated based on GGDC**.

Table S3 **Identification of bands in whole cell lysate at positions corresponding to insoluble protein bands**.

Table S4 **Cytoskeletal proteins of *S. eriocheiris* compared with other bacteria and eukaryotes**.
